# Transglutaminase 2 in cartilage homoeostasis: novel links with inflammatory osteoarthritis

**DOI:** 10.1007/s00726-016-2305-1

**Published:** 2016-08-10

**Authors:** M. Adamczyk

**Affiliations:** 1 Matrix Biology and Tissue Repair Research Unit, Oral and Biomedical Sciences, College of Biomedical and Life Sciences, School of Dentistry, Cardiff University, Heath Park, Cardiff, CF14 4XY UK; 2 Academic Unit of Bone Biology, Department of Oncology and Metabolism, Mellanby Centre For Bone Research, Medical School, The University of Sheffield, Beech Hill Road, Sheffield, S10 2RX UK

**Keywords:** Transglutaminase, Osteoarthritis, Cartilage, Inflammation, Chondrocyte hypertrophy

## Abstract

Transglutaminase 2 (TG2) is highly expressed during chondrocyte maturation and contributes to the formation of a mineralised scaffold by introducing crosslinks between extracellular matrix (ECM) proteins. In healthy cartilage, TG2 stabilises integrity of ECM and likely influences cartilage stiffness and mechanistic properties. At the same time, the abnormal accumulation of TG2 in the ECM promotes chondrocyte hypertrophy and cartilage calcification, which might be an important aspect of osteoarthritis (OA) initiation. Although excessive joint loading and injuries are one of the main causes leading to OA development, it is now being recognised that the presence of inflammatory mediators accelerates OA progression. Inflammatory signalling is known to stimulate the extracellular TG2 activity in cartilage and promote TG2-catalysed crosslinking of molecules that promote chondrocyte osteoarthritic differentiation. It is, however, unclear whether TG2 activity aims to resolve or aggravate damages within the arthritic joint. Better understanding of the complex signalling pathways linking inflammation with TG2 activities is needed to identify the role of TG2 in OA and to define possible avenues for therapeutic interventions.

## Introduction

Osteoarthritis (OA) remains the major disease causing joint degeneration and disability worldwide (Cross et al. 2014). OA is a strongly age-related disorder; however, it can affect young to middle-aged individuals or athletes and have a significant impact on people’s lives. Common symptoms of OA include: focal dysregulation within the joint, stiffness, synovial membrane swelling and pain (Goldring and Goldring 2007). The pathogenesis of OA is partly caused by the imbalance between cartilage extracellular matrix (ECM) synthesis and degradation (Goldring and Goldring 2010). The excessive mechanical joint stimulation is one of the main risk factors promoting cartilage surface thinning, erosion and loss of its elastic properties (Ko et al. 2013). The abnormal mechanical stress can lead to chondrocyte differentiation and production of cytokines, aggrecanases and matrix metalloproteinases (MMPs) which disrupt cartilage ECM (Loeser et al. 2012). Cartilage surface wear and chondrocyte apoptosis are often accompanied by subchondral bone adaptive modelling (Li et al. 2013) and synovitis (Rahmati et al. 2016). Presence of joint space narrowing, osteophytes, subchondral bone abnormalities or accumulation of the synovial fluid are the clinical features of OA (Bay-Jensen et al. 2010). The diagnosis of OA is usually confirmed by X-ray, magnetic resonance imaging or arthroscopy. Yet, these structural changes mostly detect late stages of the disease, and too little is known about the early mechanism underlying joint degeneration in OA.

Transglutaminase 2 (TG2) is an interesting target involved in OA pathogenesis as accumulation of TG2 in the ECM promotes chondrocyte hypertrophy and cartilage calcification (Tarantino et al. 2011), which mechanism might be linked to early events of the disease. TG2 belongs to the family of transglutaminases (TG), the enzymes that catalyse Ca^2+^-dependent acyl-transferase reactions (Eckert et al. 2014). Depending on the nature of the substrates available, TG mediate protein crosslinking or small amine incorporation through reaction of transamidation; peptide esterification; or catalyse hydrolysis by deamidation or isopeptide cleavage. The most characteristic reaction mediated by TG2 is that of crosslinking, which results in the formation of covalent N^ε^(γ-glutamyl)lysyl isopeptide bonds between proteins or within polypeptides (Lorand and Graham 2003). In humans, there are nine TG family members described (Eckert et al. 2014), but the main TG activity in skeletal tissues comes from the activity of TG2 and FXIIIA (Thomázy and Davies 1999; Rosenthal et al. 2001; Deasey et al. 2013). TG1 and TG3 are also detected in the skeletal tissues (Deasey et al. 2013); however, the specific biological functions of TG1 and TG3 in musculoskeletal system remain largely unknown, and this awaits further investigation. In many instances, TG2 is expressed at much higher level than any of the other enzymes of the family, thus can display various functions in disease processes (Iismaa et al. 2009). The predominant role of TG2 is in cell stress response and tissue repair, regulating hallmark events such as ECM remodelling and cell activation (Stephens et al. 2004). Yet, aberrant TG2 activity may also promote formation of protein modifications that can lead to tissue fibrosis (Verderio et al. 2004; Collighan and Griffin 2009). TG2 also drives the destructive immune responses associated with coeliac disease (Sollid and Jabri 2011) and with rheumatoid arthritis (Dzhambazov et al. 2009; Lauzier et al. 2012). Upregulation of TG2 in both OA patient samples as well as in OA animal models has been well established but the main function of TG2 in OA pathogenesis remains enigmatic. Sufficient knowledge of TG2 functions at the initial stages of OA development is needed to understand whether TG2 role is to rescue cartilage damage or promote the irreversible changes within the joint.

Inflammatory signalling stimulates TG2 extracellular crosslinking activities in articular cartilage (Johnson et al. 2001). This is important as the detection of inflammatory molecules in the OA joint in recent years has shed new light for more research into that area (de Lange-Brokaar et al. 2012; Rahmati et al. 2016). Various proinflammatory mediators were found to be elevated in the osteoarthritic joints leading to cartilage destruction, such as interleukins (IL-1β and IL-6), tumour necrosis factor α (TNFα), matrix metalloproteinases (MMP 1,3,9 and 13) and aggrecanases (ADAMTS-4 and ADAMTS-5) (Loeser et al. 2012). Tissue markers released by infiltrating macrophages or synoviocytes such as advanced glycation endproducts, high-mobility group box chromosomal protein 1 (Kyostio-Moore et al. 2011), components of the complement system (C3a and C5b-9) (Wang et al. 2012a) are indicative of “microinflammation” taking place in OA joint (Goldring and Berenbaum 2015). Thus, recent evidence suggests that OA should not be considered solely as a degenerative condition as both local inflammation and low-level systemic inflammation play a role in disease progression (Berenbaum 2012; de Lange-Brokaar et al. 2012). The presence of a novel link between TG2 and innate immunity suggests that fast accumulation of extracellular TG2 (Adamczyk et al. 2015) might occur e.g., during joint injury. This review will summarise the most recent findings about the functions of TG2 in healthy and arthritic cartilage, to highlight an important role of TG2 as a potential therapeutic target.

## TG2 expression and activity during chondrocyte maturation

TG2 is expressed and becomes activated at the early stages of long bone development (Aeschlimann and Thomazy 2000). The vast accumulation of TG2 in the cytosol of pre-hypertrophic chondrocytes is followed by TG2 secretion and catalytic activity, which is later detected in the ECM (Aeschlimann et al. 1995). The expression of TG2 diminishes when chondrocytes reach the hypertrophic stage (Aeschlimann et al. 1993). Interestingly, externalisation of TG2 from maturing chondrocytes precedes mineralisation of newly formed ECM (Aeschlimann et al. 1995). It is likely that the retinoids have a significant role in regulating TG2 expression during ossification through binding to RAR and RXR enhancer elements upstream of the *TGM2* gene (Nagy et al. 1997). TG-catalysed crosslinks stabilise structure of bone and hyaline cartilage matrix, therefore the presence of enzymatically active TG2 is considered to be an important step in long bone development (Aeschlimann et al. 1995). TG2 might also act as an autocrine factor and control glycosaminoglycans (GAGs) synthesis by regulating the xylosyltransferase 2 transcription during chondrocyte development (Nurminsky et al. 2011). In the pellet cultures of chicken mesenchymal stem cells, the upregulation of TG2 accelerates the transition of chondrocytes into the pre-hypertrophic stage and decreases deposition of extracellular matrix rich in GAGs (Nurminsky et al. 2011). Thus, TG2 plays a role in controlling matrix deposition and crosslinking chondrocyte ECM.

TG2 protein upregulation does not always correlate with an increase in TG2 catalytic activity. For example, TG2 activity is initially detected in the cartilage anlagen of the spine at E12.5 stage of mouse embryonic development, followed by the increase in the perichondrium (Itoh et al. 2013), which then slowly diminishes despite the TG2 protein expression remaining high. It is possible that the hypoxic environment of cartilage modulates TG2 levels throughout development, as hypoxia is a strong inducer of TG2 expression e.g., in cancer cells (Kumar and Mehta 2012). Clearly, more investigation is needed to underline the mechanisms controlling TG2 expression and activity during development of hyaline, elastic and fibrocartilage.

## Regulation of TG2 activity and release in chondrocytes

TG2 enzymatic activity is allosterically regulated by guanine nucleotides (GTP/GDP) and Ca^2+^ ions (Achyuthan and Greenberg 1987; Begg et al. 2006). Current understanding suggests that TG2 is rapidly converted from “closed” into the “open” conformation by high extracellular Ca^2+^ concentrations (Pinkas et al. 2007). On the other hand, GTP/GDP association promotes transition into more compact conformation (Pinkas et al. 2007). GTP is highly important as molecular manipulation of GTP-binding residues inhibits TG2 externalisation from human chondrocytes (Johnson and Terkeltaub 2005). TG2 can act as an ATPase, however, the affinity for ATP is several orders of magnitude lower compared to GTP (Achyuthan and Greenberg 1987; Schaertl et al. 2010). Hydrolysis of extracellular ATP by TG2 was postulated to be a major function of cell-surface TG2 in osteoblasts, and the hydrolysis product inorganic pyrophosphate is likely involved in promoting mineralisation (Nakano et al. 2007). Recently, it was shown that chondrocytes secrete articular cartilage matrix vesicles containing mRNA for TG2 and FXIIIA, together with ANK, type II collagen, GAPDH and aggrecan (Mitton et al. 2009). Better understanding of TG2 externalisation from chondrocytes would tell us more about its localisation and enzymatic activity in the specific domains of cartilage ECM.

## TG2–ECM interactions in articular cartilage

Chondrocyte cell surface and cartilage ECM is rich in potential binding partners for TG2. These include fibronectin (FN), collagens of type II, III, V and XI, osteopontin, osteonectin, fibrillin, laminin, syndecan-4, integrins, MMP-2 and many others (Lorand and Graham 2003; Belkin 2011). Some of the similar substrates can be targeted by another transglutaminase, FXIIIA (Muszbek et al. 2011). However, it was shown that TG2 and FXIIIA display a preference for distinct sites within the same molecular targets (Watanabe et al. 2013). In many instances, the interactions are independent of TG2 transamidation activity. For instance, TG2 binds FN at the I_6_II_1,2_I_7-9_ modules localised in the gelatin-binding region of FN, and this interaction restricts neither TG2 activity nor the interaction of FN with integrins (Gaudry et al. 1999). TG2 was also shown to associate with heparan sulphate chains of syndecan-4, without causing changes in TG2 conformation and/or affecting TG2/FN interactions (Scarpellini et al. 2009). TG2 interaction with syndecan-4 leads to protein kinase C-α activation (Telci et al. 2008) and results in inside-out signalling through β1 integrin. Although little is known about interaction of TG2 with type II collagen, it is likely that transamidation of TG2 affects the alignment of collagen fibrils and their shape as shown for corneal stroma (Wang et al. 2014). This interaction might be potentially important in mediating mechanistic properties and shear stiffness of articular cartilage.

It is likely that proteoglycans influence TG2 localisation and activity in cartilage ECM. In other connective tissues, one of the best studied examples is TG2 interaction with heparan sulphate chains on syndecan-4. Wang et al. (2012b) proposed a model, in which TG2-bound to syndecan-4 in its “closed” form is shed from the cell surface by MMPs, which leads to TG2 “opening” and re-gaining of its enzymatic activity (Wang et al. 2012b). It is, however, not known if this mechanism might be accelerated during cartilage degradation in OA. Another group identified clusters of positively charged residues on the heparan sulphate that are accessible for TG2 binding in its compact or “closed” conformation (Lortat-Jacob et al. 2012). The release of TG2 from this complex is achieved by an increase in Ca^2+^ ions, which reduces TG2 affinity for heparan sulphate (Lortat-Jacob et al. 2012), suggesting that this binding might be compromised during tissue calcification. The in vivo data showing attenuation of TG2 extracellular activity in syndecan-4^−/−^ mice indicate that excessive TG2 externalisation could be abolished by specifically cleaving heparan sulphate chains of syndecan-4 (Scarpellini et al. 2014). Thus, reduction in syndecan-4 expression might have an affect on TG2 levels in cartilage ECM due to lack of TG2 “trapping” by the heparan sulphate chains. Recently, syndecan-4 was shown to be regulating activity of ADAMTS-5 (Echtermeyer et al. 2009), which is one of the primary aggrecanases driving cartilage proteolysis in OA models (Glasson et al. 2005). Therefore, TG2 and syndecan-4 binding might reduce or promote ADAMTS-5 transactivation linked with cartilage destruction in OA. Conclusively, investigating TG2 and proteoglycan relationship in cartilage is an interesting avenue for future studies as these interactions might be important in mediating cartilage ECM fragmentation in inflammatory OA.

## Lessons from TG2^−/−^ animals

Despite the important role of TG2 in cartilage and bone development, TG2^−/−^ mice have no developmental abnormalities (De Laurenzi and Melino 2001; Nanda et al. 2001). The lack of obvious defects in TG2-deficient mice may be explained by the activity of other TGs (mainly FXIIIA) that partially compensate the absence of TG2 (see Table 1) (Deasey et al. 2013). Many studies have been conducted with use of TG2^−/−^ mice, and, collectively, these show that deficiencies become apparent once the mice are subjected to different types of insults (Oh et al. 2011). The inflammatory response in TG2^−/−^ mice is substantially different. This is manifested by an increase in IL-1β release and higher neutrophil accumulation in response to monosodium urate crystals (MSU) crystal injection (Yen et al. 2015). Also slower clearance of apoptotic neutrophils is linked to reduced ability of macrophage to effectively phagocytose cell debris (Rose et al. 2006). Similarly, there is a significant increase in number of apoptotic cells in thymus of TG2^−^/^−^ mice treated with dexamethasone and accelerated cell death of TG2^−^/^−^ thymocytes exposed to apoptotic signalling (Nanda et al. 2001). This indicates that crosslinking of cellular content by TG2 might become important in controlling cell viability and apoptotic cell clearance especially in early inflammatory processes.

**Table 1:** Compensation of TG2 expression and activity in TG2^−/−^ mice based on the original data shown by (Deasey et al. 2013). In this study, the detailed analysis of hypertrophic cartilage (knee joint), non-hypertrophic cartilage (sternum) and skeletal muscle (limb, aorta, heart, kidney and liver) was performed to compare the TG gene expression and transamidation activity between wild-type and TG2^−/−^ mice

	Wild-type mice		TG2^−/−^ mice		Compensation
	Expression	Activity	Expression	Activity	
Hypertrophic cartilage	TG2 FXIIIA TG1 (L)	60 %	FXIIIA (NC) TG1 (U) TG3 (U)	80 %	Yes (to some extent at expression and activity levels)
Non-hypertrophic cartilage	TG2 FXIIIA TG1 (L) TG3 (L)	100 %	FXIIIA (NC) TG1 (D) TG3 (ND)	3× increase	Yes (only at activity level)
Skeletal muscle	TG2 FXIIIA TG1 (L)	60 %	FXIIIA (D) TG1 (NC)	40 %	Lack

This table was prepared based on the original data published by Deasey et al. (2013)

*L* low expressed, *NC* not changed, *U* upregulated, *D* downregulated, *ND* not detected

## TG2 upregulation in OA cartilage

In the healthy knee joint, TG2 is mainly expressed in the superficial and deep zone of cartilage, as well as in the chondrocytic part of the menisci (Johnson et al. 2001). In the Hartley guinea pig model that spontaneously develops OA, TG2 expression in cartilage changes during disease progression (Huebner et al. 2009). Initially, TG2 is mainly upregulated in ECM of superficial and middle zone of cartilage, but with OA progression, it strongly accumulates in the surface layer. The enhanced formation of TG2-catalysed N^ε^(γ-glutamyl)lysyl isopeptide bonds is also evident in OA tissue (Huebner et al. 2009). Likewise, strong TG2 expression surrounding enlarged chondrocytes is detectable in cartilage collected from patients with severe OA (Johnson et al. 2001). There is a visible association between TG2 increased activity when cartilage is fibrillated and eroded (Johnson et al. 2001).

## TG2 levels and OA severity

Experiments with surgical induction of joint instability revealed that cartilage degradation is significantly reduced in TG2^−/−^ mice, and this might be important for assessing OA severity (Orlandi et al. 2009). At the same time, there is more osteophyte formation in the operated knee of TG2^−/−^ mice; therefore, lack of TG2 does not completely abolish OA-associated changes within the subchondral bone region (Orlandi et al. 2009). This may relate to differences in the inflammatory response between wild-type and TG2^−/−^ mice or be caused by higher FXIIIA activity in the lack of TG2. Human studies showed that increase in TG2 levels in the synovial fluid from OA patients correlate with the histological score of disease severity (Huebner et al. 2009). The same study also revealed that there is a positive correlation between increase in TG2 transamidation activity and meniscal degradation in OA patients above 60 years old. Remarkably, the mononuclear cells present in synovial fluid were suggested to be the source of TG2 in OA patient samples (Yen et al. 2015). Collective evidence suggests that synovial or cell-associated TG2 may be a potentially useful marker of disease progression.

## Inside-out and outside-in signalling mediated by TG2 in OA

**Fig. 1:**
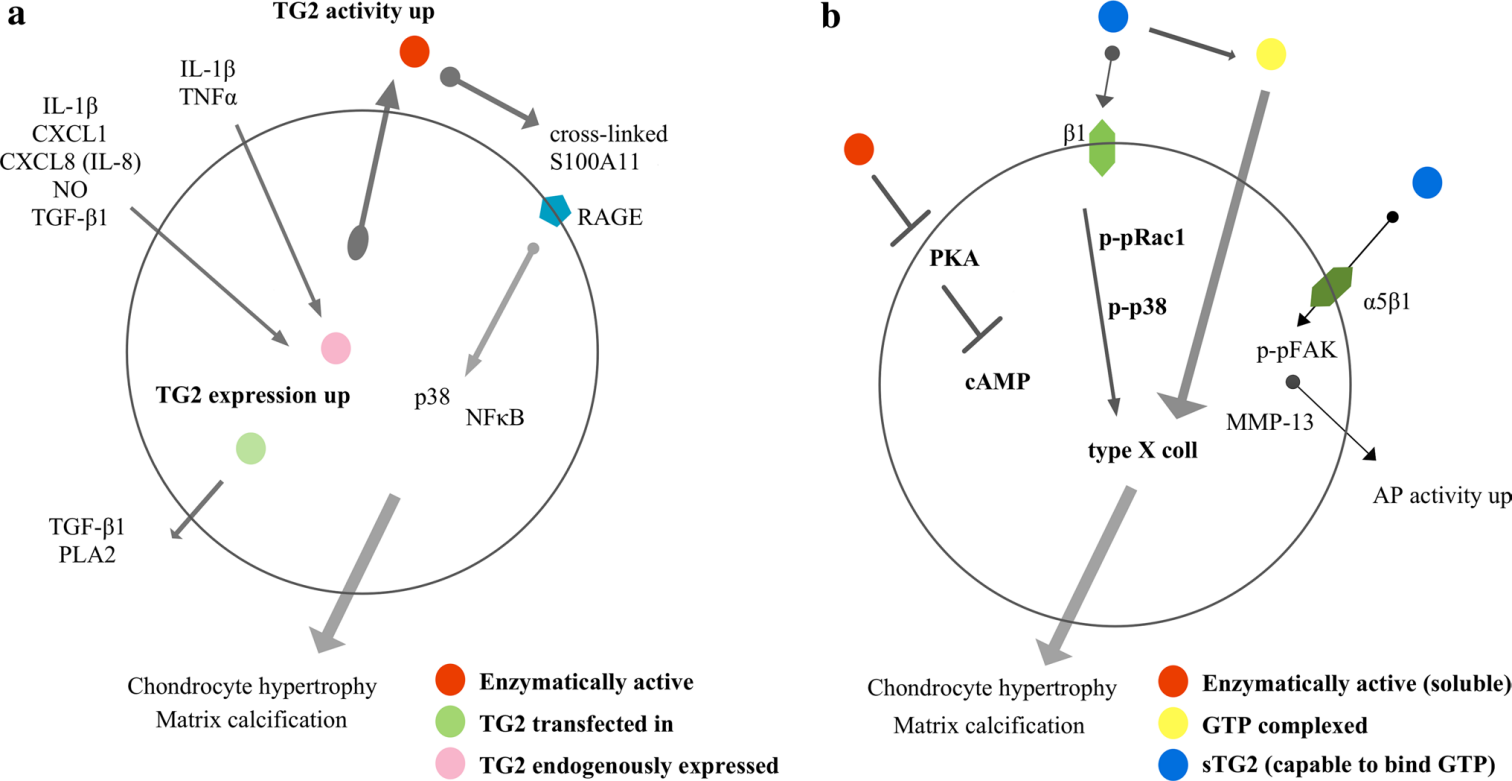
Summary of TG2-driven “*inside-out*” (**a**) and “*outside-in*” (**b**) signalling linked with chondrocyte osteoarthritic differentiation

TG2 is involved in the pathologic cartilage calcification through inside-out and outside-in signalling (Fig. 1). Mineralisation of cartilage is mediated by hypertrophic chondrocytes, which fuse and enlarge during OA progression (Goldring 2000). Treatment of chondrocytes with all-trans retinoic acid (ATRA) stimulates TG2 expression and potentiates chondrocyte re-differentiation from resting state into hypertrophic or “OA-like” chondrocytes (Johnson et al. 2001). Alongside TG2 induction, ATRA promotes expression of hypertrophic markers such as alkaline phosphatase (AP), collagen type X, or metalloproteinase-13 (MMP-13) suggestive of a direct link (Johnson et al. 2003; Huebner et al. 2009). Interestingly, transient TG2 transfection is sufficient to induce matrix calcification and collagen type X upregulation in human chondrocytes (Johnson and Terkeltaub 2005) (Fig. 1a). In meniscal chondrocytes, the activity of both TG2 and nucleoside triphosphate pyrophosphohydrolase gradually increases with age (Johnson et al. 2001). During spontaneous OA development in guinea pig, increased TG2 activity positively correlates with the increase of inorganic pyrophosphate and nucleotide pyrophosphatase phosphodiesterase (Johnson et al. 2004). Several studies demonstrated that FXIIIA transamidation activity stimulates mineralisation of osteoblast cultures (Al-Jallad et al. 2006; Nakano et al. 2010). Likely similar mechanism might be conserved in chondrocytes, whereby increase in TG2 transamidation is involved in pathological cartilage ECM calcification.

The in vitro experiments indicate that soluble TG2 (sTG2) can also act as extracellular alarmin, promoting chondrocyte osteoarthritic differentiation through outside-in signalling (Fig. 1b). Addition of sTG2 to the growth medium of human chondrocytes is sufficient to induce β1 integrin signalling that causes phosphorylation of Rac1 and p38 kinase and, ultimately, type X collagen upregulation (Johnson and Terkeltaub 2005). Moreover, the presence of soluble and enzymatically active TG2 inhibits protein kinase A (PKA) signalling and, ultimately, cyclic adenosine monophosphate (cAMP) production in chondrocytes, also causing matrix calcification (Nurminsky et al. 2011). Interestingly, the stimulation of bovine cartilage explants with sTG2 induces stronger type X collagen expression when TG2 was in complex with Mg-GTP (Johnson and Terkeltaub 2005). Thus, Johnson and Terkeltaub (2005) proposed an interesting hypothesis suggesting that pathological changes in OA may, at least in part, be due to the presence of an inactive, Mg-GTP-bound form of TG2. In agreement with those findings, stimulation of human chondrocytes with GTP complexed TG2, increases MMP-13 and type X collagen on mRNA levels (Tanaka et al. 2007). The rise in hypertrophic markers associates with elevated AP activity and possibly occurs via α5β1 integrin signalling and Fak phosphorylation. Hence, TG2, particularly in its compact conformation, might be involved in promoting hypertrophic changes and ECM calcification in cartilage.

Once released from the cells, TG2 becomes catalytically active in the extracellular space and catalyses reactions that might promote or contribute to the pathology of OA. TG2-mediated crosslinking of the S100A11 molecule allowing formation of  γ-glutamyl-ε-lysine isopeptide bridges turns it into a functional homodimer, which binds more efficiently to the cell surface receptor for advanced glycation end products (RAGE) (Cecil and Terkeltaub 2008). This enhances outside-in signalling, the activation of p38 MAPK kinase and NFκB signalling pathways, and ultimately induces chondrocyte hypertrophy.

## Regulation of TG2 by anabolic and catabolic signalling

The anabolic signalling mediated by transforming growth factor β1 (TGF-β1) is an important mechanism controlling TG2 levels in cartilage and bone. TGF-β1 seems to be a slightly better inducer of TG2 protein expression than IL-1β but has no effect on elevating TG2 activity (Johnson et al. 2001). TG2 has been proposed to have a role in TGF-β1 activation (Szondy et al. 2003), and an increased expression of TGF-β1 in TG2^−/−^ mice was reported (Tarantino et al. 2009). Therefore, TG2 in conjunction with TGF-β1 might be involved in ameliorating the inflammatory responses in the resolution phase.

The catabolic responses mediated by chemokine CXCL1 and CXCL8 (IL-8) are implicated in promoting aberrant TG2 activity (Merz et al. 2003). Also, treatment with proinflammatory IL-1β causes secretion of TG2 in mouse articular chondrocytes or bovine cartilage explants (Johnson et al. 2003; Cecil and Terkeltaub 2008). Similarly, patient-derived meniscal cells effectively upregulate TG2 activity in the presence of IL-1β (Johnson et al. 2001). It is likely that nitric oxide (NO) released upon IL-1β priming is responsible for promoting TG2 expression as peroxynitrite donors significantly enhance TG2 activity in a similar manner. TG2 seems to be the predominant TG responding to early catabolic signalling as IL-1β stimulation fails to induce transamidation activity of FXIIIA in cartilage (Johnson et al. 2003).

## Role of TG2 in gout and rheumatoid arthritis

Although the aetiology of OA is different than gout or rheumatoid arthritis, some of the mechanisms regulating TG2 might be common and exist in inflammatory type of OA. Gout is a chronic inflammatory arthritis that is characterised by the deposition of monosodium urate crystals (MSU) usually within the metatarsophalangeal, mid-foot and ankle joints (Dalbeth et al. 2016). The TG2 mRNA levels are clearly enhanced in the synovial fluid collected from gouty but not from rheumatoid arthritis patients (Yen et al. 2015). Stimulation of cells with MSU crystals alone causes an enhanced TG2 mRNA expression in primary peripheral blood monocytes or macrophages (Yen et al. 2015). It is likely that TG2 activity reduces the release of proinflammatory IL-1β and TNFα upon MSU exposure by enhancing production of anti-inflammatory TGF-β1 (Yen et al. 2015). Yet another study suggested that TG2 transamidation activity is not necessary to increase macrophage phagocytosis by TGF-β1 but be rather regulated by TG2 binding to the nucleotides, particularly GDP, ATP or ADP (Rose et al. 2006).

Interesting evidence was reported about the role of TG2 activity in rheumatoid arthritis (RA). RA is a chronic, autoimmune disease, in which synovial inflammation drives destruction of cartilage and bone by activated blood cells infiltrating joint space (Pratt et al. 2009). Induction of experimental arthritis by type II collagen immunization increases TG2 activity in the areas of cartilage erosion and within inflammed synovial membrane (Lauzier et al. 2012). The arthritic fibroblast-like synoviocytes were shown to release enzymatically active TG2 at the sites of collagen type II degradation. Importantly, TG2 was suggested to be actively involved in development of autoimmunity due to its ability to modify an immunodominant fragment of type II collagen (CII260-270) (Dzhambazov et al. 2009). TG2 crosslinking and/or deamidation of this epitope mediates a T-cell response to the CII260-270 fragment (Dzhambazov et al. 2009). Thus, TGs activity in RA animal models can enhance joint destruction by worsening the severity and histopathological features of the disease.

## Conclusions

This review demonstrates that TG2 is an interesting target in inflammatory OA pathogenesis, as there is strong evidence both from human and animal models for the involvement of TG2 in OA progression. The literature suggests that aberrant TG2 upregulation in the arthritic joint might be aggravating OA severity by promoting tissue mineralisation, accelerating chondrocyte hypertrophy and potentially enhancing OA-linked signalling. Likewise, recent findings indicate that FXIIIA might be an important player in inflammatory arthritis, as FXIIIA^−/−^ mice are protected from cartilage and bone destruction, which might be due to reduced osteoclasts fusion and activity (Raghu et al. 2015). Interestingly, treatment of arthritic joints with inhibitor cystamine to block FXIIIA activity significantly ameliorated type II collagen-induced arthritis severity in mice (Raghu et al. 2015). TG2 could also be involved as cystamine displays high specificity towards inhibiting TG2 transamination activity (Siegel and Khosla 2007). Future studies should, therefore, address TG2 functions within inflamed OA joint, taking into account the role of synovial membrane and subchondral bone into consideration despite evidence suggesting TG2 mainly as an appropriate marker of hypertrophic chondrocytes but not upregulated in the remodelled trabecular bone of OA patients (Tarquini et al. 2016). Recent findings demonstrating the role of P2X7 receptor-dependent pore formation in TG2 externalisation (Adamczyk et al. 2015) might be relevant in the context of TG2 secretion from activated synovial macrophages. This would suggest the presence of a link between innate immunity and TG2 externalisation, especially vital as P2X7 receptor activation drives the inflammatory responses in joint diseases (Labasi et al. 2002; Lopez-Castejon et al. 2010).

Managing and treating musculoskeletal diseases is a big challenge for ageing population, and there are currently no reliable biochemical markers for early patient diagnosis (Mobasheri et al. 2015). Evidence suggests that diagnostic assays based on the detection of proteins or peptides with post-translational modifications may have a higher specificity than assays detecting only changes in protein levels (Doyle and Mamula 2005). TG2 released by chondrocytes in OA could contribute to the elevated levels of γ-glutamyl-ε-lysine crosslinks present in OA tissue. In summary, more research is needed to clarify if specific post-translational modifications are generated by TG2 in certain OA phenotypes that could serve as biological indicators of the disease.
